# Supplementary material for: Appropriability and basicness of R&D: Identifying and characterising product and process inventions in patent data
Source: PLoS One. 2022 Aug 15;17(8):e0272225. doi: 10.1371/journal.pone.0272225 (PMC9377595; doi:10.1371/journal.pone.0272225)
Supplement: S1 File — This appendix describes the implementation of the keyword search, the identification of independent and product-by-process claims, and the construction of a measure for growing technologies. (PDF) [file pone.0272225.s001.pdf]

S1 File: Appendix

Implementation of the keyword search

In order to implement the keyword search, we defined additional heuristic rules to exploit the structure of the claims. In order to apply the rules, we had to pre-process the raw claim texts: We defined a set of stop words that we removed from the raw claim texts, we removed all accents and umlauts, all numbers, and set everything to lower cases. The most important rule says: Restrict the keyword search to the first two or five words of a processed claim text in order to identify process claims and restrict the keyword search to the first word of a preprocessed claim text in order to identify use claims. This was necessary because otherwise we would have classified many product claims as processes by mistake (for example “The toner (. . .), wherein, in a distribution of particle diameter measured by a Coulter method, the content of large grains having a particle diameter of 8 μm or more is 2% by mass or less.”, patent number EP2423755B1).

In most cases, the process keyword comes right at the beginning of the claim, (e.g., ”The method of manufacturing an SOI substrate according to Claim 3, wherein the second insulating film is formed of a single-layer structure formed of a single layer or a stacked-layer structure formed of a plurality of layers (. . .).”, patent number EP1986230A2). In rare cases, other words precede the process keyword, (e.g., ”The digital rights method of claim 2, wherein modifying the existing digital rights comprises setting the DRM method to the Forward-Lock method if the DRM methods applied to the digital rights and existing digital rights are different, (. . .).”, patent number EP1942429A2). The thresholds of two resp. five words were chosen after inspecting thousands of claims manually and they apply to processed claim texts where articles preceding the keywords have been removed. In the end, for aggregate indicators at patent or firm level it does not make a significant difference whether we use two or five words. For use keywords, we could not find any claims where other words precede the keyword after having removed the stop words. That is why we identify use claims based on the first word. We always searched for both the singular (‘method’) and plural (‘methods’ or ‘methoden’ in German).

In a final step, we imputed missing EPO (USPTO) claim information from within the family by prioritizing information from other EPO (USPTO) patents over USPTO (EPO) patents because claims from another EPO (USPTO) publication should be closer to the focal EPO (USPTO) patent than a USPTO (EPO) publication.

Identification of independent and dependent claims

The difference between independent and dependent claims is that a dependent claim cannot stand alone, this means it references another claim (independent claim) that is directed to the essential features of the invention (e.g., “The method of manufacturing an SOI substrate according to Claim 3 (. . .)”, patent number EP1986230A2). The distinction might play a role for aggregated claim-based indicators. From an economic point of view, an applicant wants to include as many claims as possible in order to increase the patent’s breadth. Measures only based on independent claims might therefore have the advantage of considering only “relevant” product and process features

and filtering out content that has been added for strategic reasons. We therefore provide all measures in our database also calculated based on only independent claims.

The most common phrases used in dependent claims are ‘according to [independent claim]’, ‘according to (any) one of the preceding claims’, ‘as claimed in [independent claim]’, ‘in accordance with [independent claim]’. The identification of dependent claims is easy: Whenever the word ‘claim’ or ‘claims’ appears in the claim text but not in the first word, it must be a dependent claim. Accordingly, all other claims have been classified as independent claims. We also applied this strategy on all claims in German and French using the respective keywords in German and French (’anspruch’, ’anspruche’, ‘anspruech’, ’anspruech e’, ’anspruchen’, ’anspruechen’, ‘revendication’, ’revendications’).

Identification of product-by-process claims

A product-by-process claim defines a product in terms of a new process (for example, “an article A, characterized by being the product of process B“, or “an article A obtained by process B”). Product-by-process claims are product claims. We need to classify them separately because otherwise we would run the risk of classifying them as process claims based on the process keywords appearing in the same claim.

The typical product-by-process claim includes words such as “according to [process keyword]”, “obtainable by [process keyword]”, “produced by [process keyword]”, “purified from [process keyword]”, “the product of the [process keyword] comprising the steps of”, “prepared in accordance with the [process keyword]”, “by a [process keyword] which comprises the steps of” [1]. We checked a random sample of claims for further phrases indicating product-by-process claims. We are confident that we have identified the bulk of phrases so that we can classify most of the product-by-process claims correctly. We applied an exclusion strategy to increase the likelihood of true positives: We did not allow for any of the process (or use) keywords from Table **??** to show up in the first two words of the pre-processed claim text. Thus, we made sure that a product label must appear in the first two words. In addition, we searched for all extracted phrases which need to be followed by a process keyword (for example, the product showing up at the beginning has to be produced ‘according to’ or ‘obtainable from’ a process). We also run the code with phrases and keywords in German and French.

Identification of growing technologies

We constructed a unique variable that measures for each patent application whether it was filed in the upward slope of a life cycle or in the downward slope that might coincide with the end of a technological life cycle. We started by querying all possible combinations of IPC subclasses available in PATSTAT, while many patents cover more than one IPC subclass. To give an example, the patent application EP2355317A1 filed by the Siemens AG has been assigned to H02M 1/12, H02M 5/458, and H02P 21/05. Subclasses cover the first four digits of the IPC so that the patent application belongs to the subclass combination H02M, H02P (Electric machines not otherwise provided for, Control or regulation of electric motors electric generators or dynamo-electric converters; controlling transformers, reactors or choke coils). In sum, we could find 119370 combinations of IPC subclasses in PATSTAT. For each combination, we applied a kernel-weighted local polynomial regression of the number of patent applications at the USPTO and EPO on filing year and stored the smoothed values y. We calculated the difference between the smoothed number of patent applications in year t and year t-1 for each combination and created a variable with value -1 if the difference is

negative, +1 if it is positive, and 0 if it is zero. This indicator allows us to determine for each combination and year if the difference of the number of patent applications shows an upward or downward trend. In addition, we can investigate how old each technological combination is by looking at the first patent application that has been filed in each combination.

References

1. Chang BC, Wang SJ. The limited benefit of "product-by-process" claim. Human vaccines & immunotherapeutics. 2016;12(10):2685–2687.
