# Supplementary material for: Appropriability and basicness of R&D: Identifying and characterising product and process inventions in patent data
Source: PLoS One. 2022 Aug 15;17(8):e0272225. doi: 10.1371/journal.pone.0272225 (PMC9377595; doi:10.1371/journal.pone.0272225)
Supplement: S1 Table — These tables contain the results from the multinomial logit model with different base categories. (PDF) [file pone.0272225.s002.pdf]

S1 Table: Regression results with different basis

Table 1. Multinomial logit reduced model - dependent variable: patent category, base category: process patent.

	categorization based on all claims		categorization based on only independent claims	
	product patent (1)	mixed patent (2)	product patent (3)	mixed patent (4)
Citations	-0.0877***	0.194***	-0.0355***	0.196***
	(0.00212)	(0.00207)	(0.00196)	(0.00214)
Originality and Radicalness	-0.113***	-0.0124***	-0.108***	0.00183
	(0.00172)	(0.00181)	(0.00161)	(0.00184)
Age and Scope	-0.109***	-0.0155***	-0.0985***	-0.0353***
	(0.00175)	(0.00180)	(0.00162)	(0.00185)
Claims	-0.0332***	-0.694***	-0.101***	-1.254***
	(0.00156)	(0.00204)	(0.00154)	(0.00217)
USPTO	-0.0647***	-0.924***	-0.00450	-0.717***
	(0.00462)	(0.00485)	(0.00406)	(0.00485)
Constant	0.677***	1.305***	0.572**	0.720***
	(0.195)	(0.184)	(0.178)	(0.199)
Observations	4800465		4800110	
Pseudo R^2	0.186		0.204	
chi2	1259919.8		1343087.3	

Robust standard errors in parentheses. * $p < 0.05$, ** $p < 0.01$, *** $p < 0.001$. Time dummies, inventor country and technology shares included.

Table 2. Multinomial logit reduced model - dependent variable: patent category, base category: mixed patent.

	categorization based on all claims		categorization based on only independent claims	
	product patent (1)	process patent (2)	product patent (3)	process patent (4)
Citations	-0.282***	-0.194***	-0.231***	-0.196***
	(0.00149)	(0.00207)	(0.00155)	(0.00214)
Originality and Radicalness	-0.101***	0.0124***	-0.110***	-0.00183
	(0.00120)	(0.00181)	(0.00131)	(0.00184)
Age and Scope	-0.0939***	0.0155***	-0.0632***	0.0353***
	(0.00130)	(0.00180)	(0.00141)	(0.00185)
Claims	0.660***	0.694***	1.153***	1.254***
	(0.00160)	(0.00204)	(0.00183)	(0.00217)
USPTO	0.859***	0.924***	0.713***	0.717***
	(0.00329)	(0.00485)	(0.00372)	(0.00485)
Constant	-0.628***	-1.305***	-0.147	-0.720***
	(0.160)	(0.184)	(0.168)	(0.199)
Observations	4800465		4800110	
Pseudo R^2	0.186		0.204	
chi2	1259919.8		1343087.3	

Robust standard errors in parentheses. * $p < 0.05$, ** $p < 0.01$, *** $p < 0.001$. Time dummies, inventor country and technology shares included.

Table 3. Multinomial logit full model - dependent variable: patent category, base category: process patent.

	categorization based on all claims		categorization based on only independent claims	
	product patent (1)	mixed patent (2)	product patent (3)	mixed patent (4)
Citations	-0.0691***	0.206***	-0.0228***	0.203***
	(0.00217)	(0.00213)	(0.00201)	(0.00220)
Originality and Radicalness	-0.112***	-0.0196***	-0.109***	-0.00708***
	(0.00172)	(0.00181)	(0.00161)	(0.00184)
Age and Scope	-0.0955***	-0.00806***	-0.0889***	-0.0346***
	(0.00183)	(0.00189)	(0.00169)	(0.00194)
Claims	-0.0283***	-0.681***	-0.0973***	-1.242***
	(0.00156)	(0.00205)	(0.00155)	(0.00218)
USPTO	-0.0835***	-0.934***	-0.0122**	-0.708***
	(0.00478)	(0.00503)	(0.00422)	(0.00506)
upward_tech_cycle	0.0648***	0.0582***	0.0561***	0.0566***
	(0.00236)	(0.00245)	(0.00218)	(0.00255)
lnfamily_size	-0.00263	-0.0219***	0.00493*	0.0131***
	(0.00236)	(0.00239)	(0.00220)	(0.00248)
lnnb_inventors	-0.251***	-0.0509***	-0.182***	-0.0910***
	(0.00392)	(0.00403)	(0.00366)	(0.00415)
lnnumb_words_ind	-0.0513***	-0.521***	-0.135***	-0.680***
	(0.00438)	(0.00440)	(0.00402)	(0.00456)
Constant	1.552***	3.264***	1.515***	3.218***
	(0.196)	(0.186)	(0.179)	(0.203)
Observations	4800271		4799916	
Pseudo R^2	0.189		0.208	
chi2	1272444.8		1350628.1	

Robust standard errors in parentheses. * $p < 0.05$, ** $p < 0.01$, *** $p < 0.001$. Time dummies, inventor country and technology shares included.

Table 4. Multinomial logit full model - dependent variable: patent category, base category: mixed patent.

	categorization based on all claims		categorization based on only independent claims	
	process patent (1)	mixed patent (2)	process patent (3)	mixed patent (4)
Citations	-0.275***	-0.206***	-0.226***	-0.203***
	(0.00152)	(0.00213)	(0.00160)	(0.00220)
Originality and Radicalness	-0.0923***	0.0196***	-0.102***	0.00708***
	(0.00121)	(0.00181)	(0.00132)	(0.00184)
Age and Scope	-0.0874***	0.00806***	-0.0544***	0.0346***
	(0.00135)	(0.00189)	(0.00146)	(0.00194)
Claims	0.652***	0.681***	1.145***	1.242***
	(0.00161)	(0.00205)	(0.00184)	(0.00218)
USPTO	0.851***	0.934***	0.696***	0.708***
	(0.00343)	(0.00503)	(0.00388)	(0.00506)
upward_tech_cycle	0.00653***	-0.0582***	-0.000445	-0.0566***
	(0.00180)	(0.00245)	(0.00197)	(0.00255)
lnfamily_size	0.0193***	0.0219***	-0.00813***	-0.0131***
	(0.00173)	(0.00239)	(0.00185)	(0.00248)
lnnb_inventors	-0.200***	0.0509***	-0.0915***	0.0910***
	(0.00288)	(0.00403)	(0.00311)	(0.00415)
lnnumb_words_ind	0.470***	0.521***	0.545***	0.680***
	(0.00354)	(0.00440)	(0.00371)	(0.00456)
Constant	-1.712***	-3.264***	-1.703***	-3.218***
	(0.161)	(0.186)	(0.169)	(0.203)
Observations	4800271		4799916	
Pseudo R^2	0.189		0.208	
chi2	1272444.8		1350628.1	

Robust standard errors in parentheses. * $p < 0.05$, ** $p < 0.01$, *** $p < 0.001$. Time dummies, inventor country and technology shares included.
